# Salidroside Improves Behavioral and Histological Outcomes and Reduces Apoptosis via PI3K/Akt Signaling after Experimental Traumatic Brain Injury

**DOI:** 10.1371/journal.pone.0045763

**Published:** 2012-09-24

**Authors:** Szu-Fu Chen, Hsin-Ju Tsai, Tai-Ho Hung, Chien-Cheng Chen, Chao Yu Lee, Chun-Hu Wu, Pei-Yi Wang, Nien-Chieh Liao

**Affiliations:** 1 Departments of Physiology and Biophysics, National Defense Medical Center, Taipei, Taiwan, Republic of China; 2 Department of Physical Medicine and Rehabilitation, Cheng Hsin General Hospital, Taipei, Taiwan, Republic of China; 3 Department of Obstetrics and Gynecology, Chang Gung Memorial Hospital at Taipei and College of Medicine, Chang Gung University, Taipei, Taiwan, Republic of China; 4 Department of Neuroscience, Institute of Psychiatry, King’s College London, London, United Kingdom; 5 Department of Clinical Pathology, Cheng Hsin General Hospital, Taipei, Taiwan, Republic of China; St. Jude Children's Research Hospital, United States of America

## Abstract

**Background:**

Traumatic brain injury (TBI) induces a complex sequence of apopototic cascades that contribute to secondary tissue damage. The aim of this study was to investigate the effects of salidroside, a phenolic glycoside with potent anti-apoptotic properties, on behavioral and histological outcomes, brain edema, and apoptosis following experimental TBI and the possible involvement of the phosphoinositide 3-kinase/protein kinase B (PI3K)/Akt signaling pathway.

**Methodology/Principal Findings:**

Mice subjected to controlled cortical impact injury received intraperitoneal salidroside (20, or 50 mg/kg) or vehicle injection 10 min after injury. Behavioral studies, histology analysis and brain water content assessment were performed. Levels of PI3K/Akt signaling-related molecules, apoptosis-related proteins, cytochrome C (CytoC), and Smac/DIABLO were also analyzed. LY294002, a PI3K inhibitor, was administered to examine the mechanism of protection. The protective effect of salidroside was also investigated in primary cultured neurons subjected to stretch injury. Treatment with 20 mg/kg salidroside_significantly improved functional recovery and reduced brain tissue damage up to post-injury day 28. Salidroside_also significantly reduced neuronal death, apoptosis, and brain edema at day 1. These changes were associated with significant decreases in cleaved caspase-3, CytoC, and Smac/DIABLO at days 1 and 3. Salidroside increased phosphorylation of Akt on Ser473 and the mitochondrial Bcl-2/Bax ratio at day 1, and enhanced phosphorylation of Akt on Thr308 at day 3. This beneficial effect was abolished by pre-injection of LY294002. Moreover, delayed administration of salidroside at 3 or 6 h post-injury reduced neuronal damage at day 1. Salidroside treatment also decreased neuronal vulnerability to stretch-induced injury *in vitro*.

**Conclusions/Significance:**

Post-injury salidroside improved long-term behavioral and histological outcomes and reduced brain edema and apoptosis following TBI, at least partially via the PI3K/Akt signaling pathway.

## Introduction

Traumatic brain injury (TBI) triggers a complex cascade of apoptotic events that cause delayed tissue damage [1]. Initiation of apoptosis may occur through oligomerization of cell surface death receptors, the “extrinsic” pathway, or by mitochondrial signals, the “intrinsic” pathway [1]. Clinically, cleavage of caspase-1, caspase -3, and caspase 8 were found in brain tissue of TBI patients, suggesting activation of caspase-dependent apoptosis [2], [3]. Studies in patients have also shown an increase in the levels of various apoptotic markers in both cerebrospinal fluid (CSF) and surgically resected tissue after TBI [2], [4], [5]. Furthermore, DNA fragmentation in cells with apoptotic morphologies has been reported in post-mortem analysis of brain tissue of patients surviving up to 12 months post-TBI [6]. This delayed apoptosis provides a relatively wide therapeutic window for intervention.

Intrinsic apoptosis is caused by the release of mitochondrial intermembrane space (IMS) proteins, such as cytochrome C (CytoC) and Smac/DIABLO, into the cytosol, leading to subsequent caspase activation [1]. The translocation of pro-apoptotic molecules, Bax and Bak, to the mitochondria is considered to be the main mechanism contributing to mitochondrial dysfunction. On the other hand, the anti-apoptotic survival factors such as Bcl-2 can halt apoptosis by inhibiting Bax from undergoing conformational changes and forming higher-order oligomers onto the mitochondrial membrane, thus stabilizing mitochondrial integrity [7]. The Bax/Bcl-2 ratio has been proposed to be a rheostat that regulates apoptosis [8]. The “extrinsic pathway” is triggered by ligands binding to plasma membrane “death receptors” (tumor necrosis factor [TNF] receptor and Fas receptor) [1]. Activated death receptors form complexes with intracellular signaling molecules and procaspase-8, resulting in procaspase-8 auto-cleavage and activation. Caspase-8 cleaves caspase-3 to its activated form, causing activation of Bid to truncated Bid (tBid), which amplifies intrinsic apoptosis via activation of Bax. These studies suggest the convergence of 2 death pathways on mitochondria.

While the injured brain activates several damaging processes to induce apoptosis, it concomitantly triggers self-protective mechanisms to counteract tissue damage and promote neuronal survival [9]. The phosphoinositide-3-kinase (PI3K)/Akt signaling pathway plays a crucial role in regulating cell survival. Activation of Akt involves phosphorylation on both Thr308 and Ser473, and then p-Akt functions through its kinase activity. Activated Akt phosphorylates several downstream target proteins, including Bad and forkhead box O (FOXO) to prevent apoptosis [10]. p-Akt phosphorylates the death promoter Bad to maintain mitochondrial integrity by preventing the inhibition of anti-apoptotic Bcl-2 by Bad. In addition, p-Akt blocks Fas ligand transcription by phosphorylating FOXO, thereby interfering with ligand-induced extrinsic apoptosis [11]. These studies suggest that pharmacological activation of Akt signaling may be a useful strategy for protection of the injured brain.

Salidroside, a compound with the chemical structure of phenol glycosides, is a potent antioxidant extracted from the root of *Rhodiola rosea.* This traditional Tibetan medicine was used as an adaptogen to enhance the body’s resistance to fatigue by athletes and pilots. Apart from roles in anti-inflammation [12] and anti-oxidation [13], salidroside has been shown to exert potent anti-apoptotic effects in various cell types and disease models, including neurons [13], cardiomyocytes [14], endothelia [15], and acute myocardial infarction in rats [14]. In this regard, salidroside upregulated survival signals, such as the Bcl-2/Bax ratio and Akt phosphorylation, and maintained mitochondrial integrity [14]–[17]. Increasing evidence suggests that salidroside may have neuroprotective effects in the injured brain. *In vitro* studies have shown that salidroside protects against neuronal apoptotic death induced by various stimuli, such as glutamate [18], H_2_O_2_ [19], and hypoglycemia/serum limitation [20], mechanisms mimicking secondary injury cascades in TBI. Salidroside also attenuated early ischemic brain injury and improved acute behavioral dysfunctions caused by focal cerebral ischemia [21]. Despite evidence indicating the prophylactic effects of salidroside treatment on early neurological recovery in the stroke model, no information is available concerning the long-term effects of salidroside on functional recovery or tissue preservation in the injured brain. In particular, the therapeutic efficacy of salidroside administered after acute TBI has not been established.

The aim of the present study was to investigate the protective effects of salidroside on apoptosis, brain edema, and long-term behavioral outcomes after TBI. We further examined whether salidroside could enhance the PI3K/Akt survival signaling, thereby reducing brain damage.

## Results

### Cellular Localization of p-Akt Signaling following TBI

**Figure 1:**
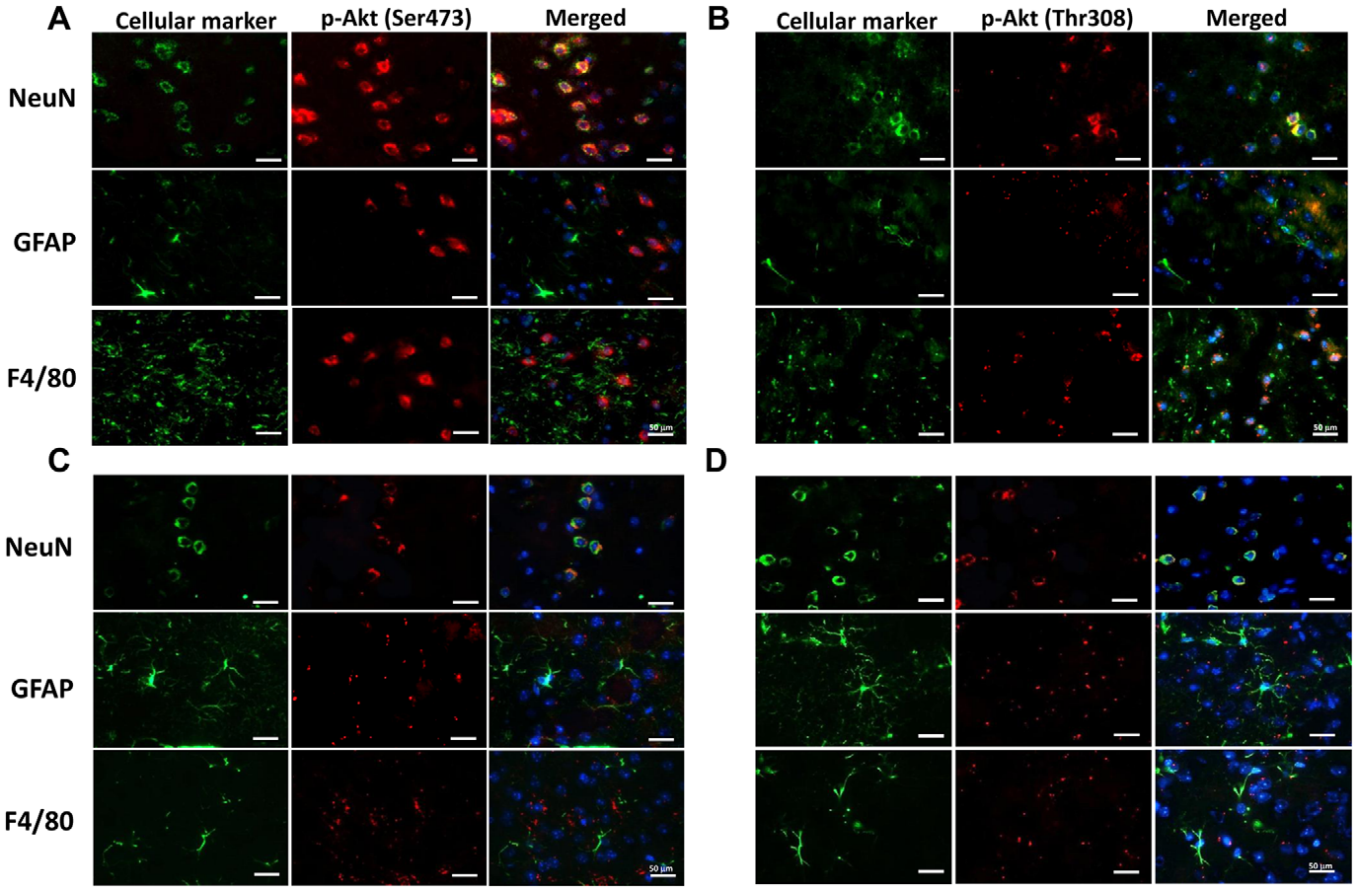
Cell distribution of p-Akt following controlled cortical impact (CCI) at day 1 post-injury. Cellular localization of (**A**) p-Akt Ser473, (**B**) p-Akt Thr308 in the peri-contusion margin, and (**C**) p-Akt Ser473 and (**D**) p-Akt Thr308 in the contralateral hemisphere 1 day post-injury, observed by immunofluorescence labeling. p-Akt immunoreactivity is shown in red, and immunolabeling of NeuN (a cell marker for neurons), F4/80 (a cell marker for microglia), or GFAP (a cell marker for astrocytes) is shown in green. Yellow labeling indicates co-localization. Both p-Akt Ser473 and Thr308 were mainly expressed in neurons, but rarely in astrocytes or microglia. Sections were stained with DAPI (blue) to show all nuclei. The scale bar is 50 µm.

First, we investigated the localization of the survival signal p-Akt following controlled cortical impact (CCI) injury in mice. The immune-positive signals of both p-Akt Ser473 and Thr308 were predominantly found in the cortical areas around the impact site. Immunofluorescent double staining revealed that both p-Akt Ser473 and Thr308 were mainly expressed in neurons, but rarely in astrocytes or microglia (Figs. 1A and B). Immunofluorescent analysis of the contralateral hemisphere of injured mice showed results identical to that of the ipsilateral hemisphere (Figs. 1C and D).

### Post-injury Salidroside Treatment Improved Long-term Functional Recovery and Attenuated Brain Edema

In order to assess the protective efficacy of endogenous survival signals in CCI, we used salidroside to activate PI3K/Akt signaling following CCI. Treatment with 20 mg/kg salidroside (SALD 20) did not alter plasma concentrations of blood urea nitrogen (BUN), creatinine (CRE), or alanine aminotransferase (ALT) (Table 1). The mice in all groups lost weight (∼6%) during the initial 4 days after CCI, but their weight normalized within 7 days. No significant difference in body weight was detected among groups treated with SALD 20, 50 mg/kg salidroside (SALD 50), or vehicle (*P*>0.05, data not shown).

**Table 1:** Metabolic characteristics of the sham control, mice treated with saline and salidroside.

	Sham	Saline	SALD 20 mg/kg	SALD 50 mg/kg	Reference range
**ALT (mg/dL)**	18.75±1.31	19.17±3.49	22.16±3.65	20.38±0.50	35
**BUN (mg/dL)**	27.00±2.19	24.32±0.68	23.2±2.49	28.06±4.5	17–28
**CRE (mg/dL)**	0.13±0.01	0.15±0.01	0.17±0.01	0.16±0.04	0.3–1.0

Values are expressed as means ± SEM. n = 4–5 mice/group. BUN: blood urea nitrogen; CRE: creatinine; ALT: alanine aminotransferase.

**Figure 2:**
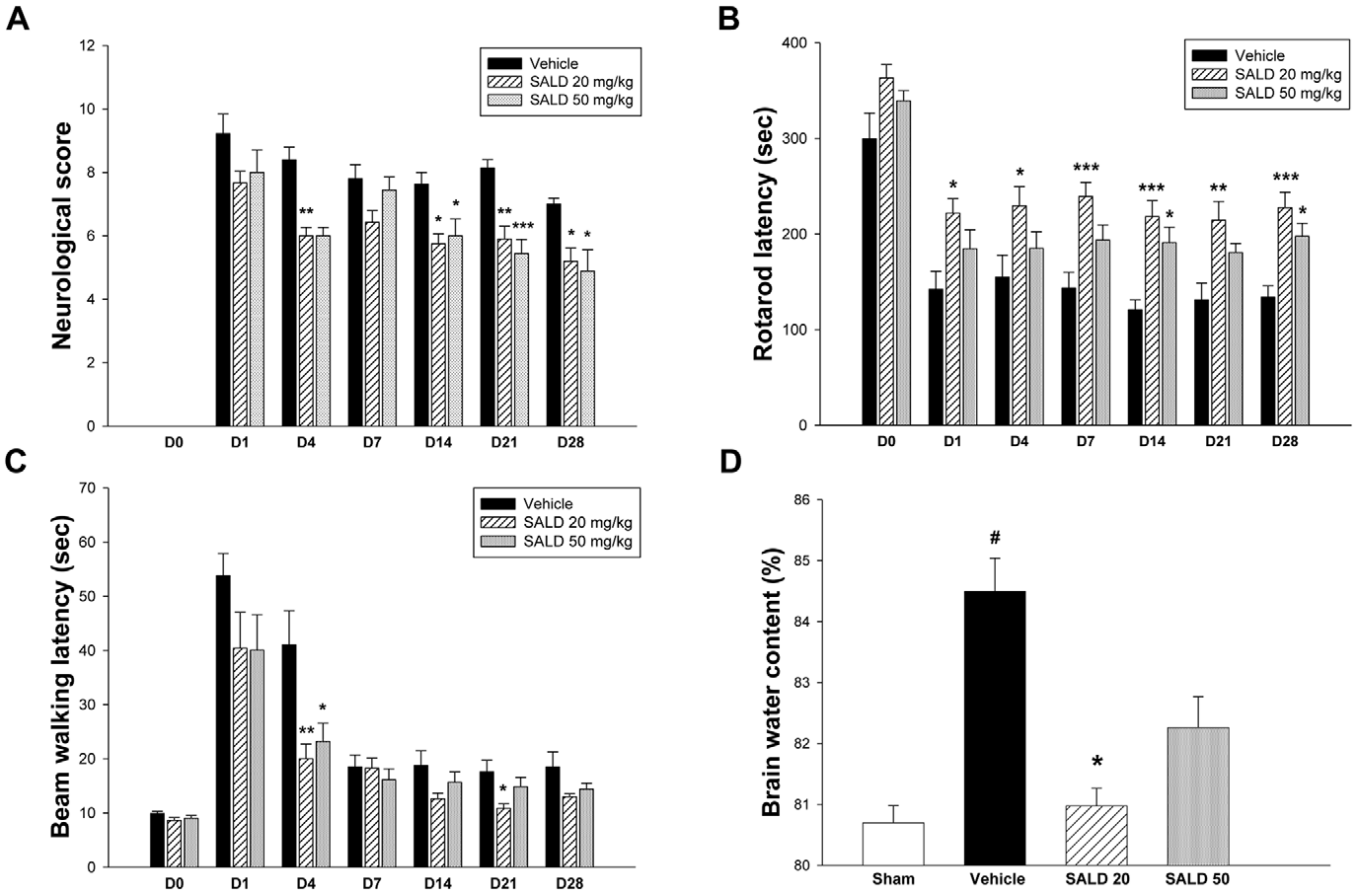
Effects of different doses of salidroside on behavioral outcomes and brain edema in contusion-injured mice. (**A**) Both 20 mg/kg salidroside (SALD 20) and 50 mg/kg salidroside (SALD 50) treatments reduced modified neurological severity score (mNSS), and the mNSS scores in the SALD 20 group were significantly lower compared with the corresponding vehicle group at days 4, 14, 21, and 28 (all *P*<0.05). The SALD 50 group had significantly lower mNSS scores from day 14 (*P*<0.05 on days 14, 21 and 28). (**B**) Both SALD 20 and SALD 50 groups had better rotarod performance compared to the vehicle group. The SALD 20 group had significantly better performance over the whole observation period (all *P*<0. 05), and SALD 50 significantly improved the rotarod outcome at days 14 and 28 (both *P*<0. 05). (**C**) There was significant difference between the SALD 20 and the vehicle groups at days 4 and 21 (*P*<0.05 and 0.01, respectively) for the beam walk latency. The treatment effect was significant only at day 4 for the SALD 50 group (*P*<0. 05). Values are presented as mean ± SEM; *P<0.05, **P<0.01, ***P<0.001 versus vehicle-treated injured mice. (n = 8–10 mice/group at each time point for behavior tests, repeated measures two-way ANOVA). (**D**) SALD 20 treatment reduced brain water content in the ipsilateral hemisphere compared to the vehicle group at day 1. Values are presented as mean ± SEM; **^#^** *P*<0.05 versus sham controls, and *P<0.05 versus vehicle-treated injured mice (n = 6 mice/group for brain water content, one-way ANOVA).

We first conducted several sets of behavioral experiments to verify whether post-injury salidroside treatment could improve recovery from neurological deficits. Neurological deficits were observed after injury, and these deficits were itemized and quantified as modified neurological severity scores (mNSS) (Fig. 2A). Both SALD 20 and SALD 50 treatments reduced the mNSS scores, and the mNSS scores in the SALD 20 group were significant lower than those in the corresponding vehicle group at days 4, 14, 21, and 28 (all *P*<0.05). The SALD 50 group had significantly lower mNSS scores from day 14 (*P*<0.05 on days 14, 21, and 28). Mice subjected to CCI presented significant motor dysfunction, as assessed by rotarod and beam walking tests. Both SALD 20 and SALD 50 groups had better rotarod performance compared to that of the vehicle group, particularly the SALD 20 group, which had significantly better performance over the whole observation period (all *P*<0. 05, Fig. 2B). SALD 50 significantly improved the rotarod outcome only at days 14 and 28 (*P*<0. 05). Likewise, beam walk latencies were shorter for both the SALD 20 and SALD 50 groups, and there was significant difference between the SALD 20 and the vehicle groups at days 4 and 21 (*P*<0.05 and 0.01, respectively; Fig. 2C). The treatment effect was significant only at day 4 for the SALD 50 group (*P*<0. 05).

Brain edema was evaluated at day 1, when brain edema is reported to reach the maximum following CCI in mice [22]. Brain water content, an indicator of brain edema, was significantly increased in the ipsilateral hemisphere by day 1 in the vehicle group compared to that of the sham-operated group (84.5±1.2% versus 80.7±0.6%; *P*<0.05; Fig. 2D). Treatment with SALD 20 caused a reduction in the percentage of water content within the ipsilateral hemisphere cortex compared with the vehicle group (81.0±0.6% versus 84.5±1.2%; *P*<0.05; Fig. 2D). There was no significant difference between the SALD 50 and vehicle groups (82.2±1.1% versus 84.5±1.2%).

### Post-injury Salidroside Treatment Reduced Brain Tissue Loss and Neuronal Damage

**Figure 3:**
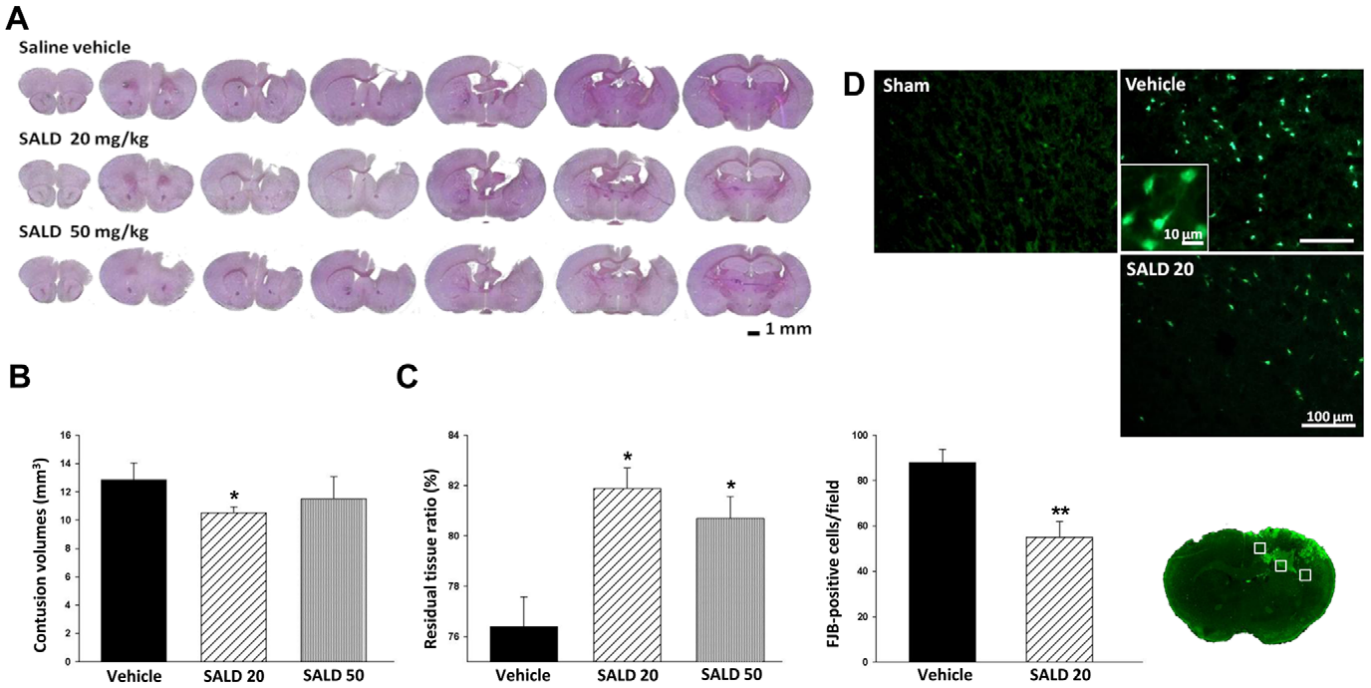
Effects of different doses of salidroside on histological outcomes in contusion-injured mice. (**A**) Representative cresyl violet-stained brain sections of vehicle-treated, 20 mg/kg (SALD 20) and 50 mg/kg salidroside (SALD 50)-treated mice 28 days post-injury, showing a pronounced loss of tissue in the injured hemisphere. Scale bar is 1 mm. (**B**) Quantification revealed significantly smaller contusion volumes in SALD 20-treated mice compared with vehicle-treated mice at day 28. (**C**) Both SALD 20 and SALD 50 caused significantly greater preservation of brain tissue compared with the vehicle group at day 28. (**D**) A representative Fluoro-Jade B (FJB)-stained brain section of a core contusional region at 0.74 mm from the bregma at day 1. Quantification analysis indicated that SALD 20-treated mice had significantly fewer degenerating neurons than vehicle-treated mice in the cortical contusion margin at day 1 post-injury. The total number of FJB-positive cells is expressed as the mean number per field of view (0.8 mm^2^). The scale bar is 50 µm. Values are presented as mean ± SEM; *P<0.05, **P<0.01 versus vehicle-treated injured mice (n = 9 mice/group for cresyl-violet staining, one-way ANOVA and 5 mice/group for FJB histochemistry, Student’s *t*-test).

Since both SALD 20 and SALD 50 treatments significantly improved functional recovery, we then investigated whether these treatment paradigms ameliorated brain tissue loss and neuronal damage. CCI induced a pronounced loss of tissue in the injured hemisphere at post-injury day 28 (Fig. 3A). Contusion volume in the vehicle group was 13.5±4.0 mm^3^. SALD 20 treatment significantly reduced contusion volume to 73.8% of the vehicle group and was 9.9±1.1 mm^3^ (*P*<0.05); however, there was no significant change in contusion volume following SALD 50 treatment (11.7±3.2 mm^3^; *P*>0.05 versus vehicle group; Fig. 3B). At day 28, the ipsilateral cortex and striatum were notably more atrophied than the contralateral side, indicating tissue loss in the ipsilateral hemisphere. Treatment with both SALD 20 and SALD 50 resulted in significantly greater preservation of brain tissue (81.9±2.6% for SALD 20 and 80.7±2.8% for SALD 50) compared to the vehicle group (76.4±3.3%) (both *P*<0. 05; Fig. 3C). However, there was no significant difference between the SALD 20 and SALD 50 groups (*P*>0.05).

Since SALD 20 significantly improved functional recovery, and attenuated brain tissue loss and cerebral edema, we used this dosage to examine the effect of SALD further. The tissue loss in long-term observation has been shown to be associated with neurodegeneration during the acute phase [23], so we analyzed neuronal degeneration in injured brain using Fluoro-Jade B (FJB) histochemistry at day 1 post-injury. The day 1 time point was chosen because FJB reactivity has been shown to peak 1 day after CCI [24]. Prominent FJB staining was observed in the ipsilateral cortex and striatum but not in the contralateral hemisphere in both the SALD 20 and vehicle groups (Fig. 3D). SALD 20 treatment significantly reduced the number of FJB-positive neurons around the injured cortical area compared to the vehicle group (55.1±6.9 versus 87.9±5.9 cells/field; *P*<0.01; Fig. 3D).

### Post-injury SALD Treatment Reduced Apoptotic Cell Death and Inhibited the Release of Mitochondrial Inner Proteins into the Cytosol

**Figure 4:**
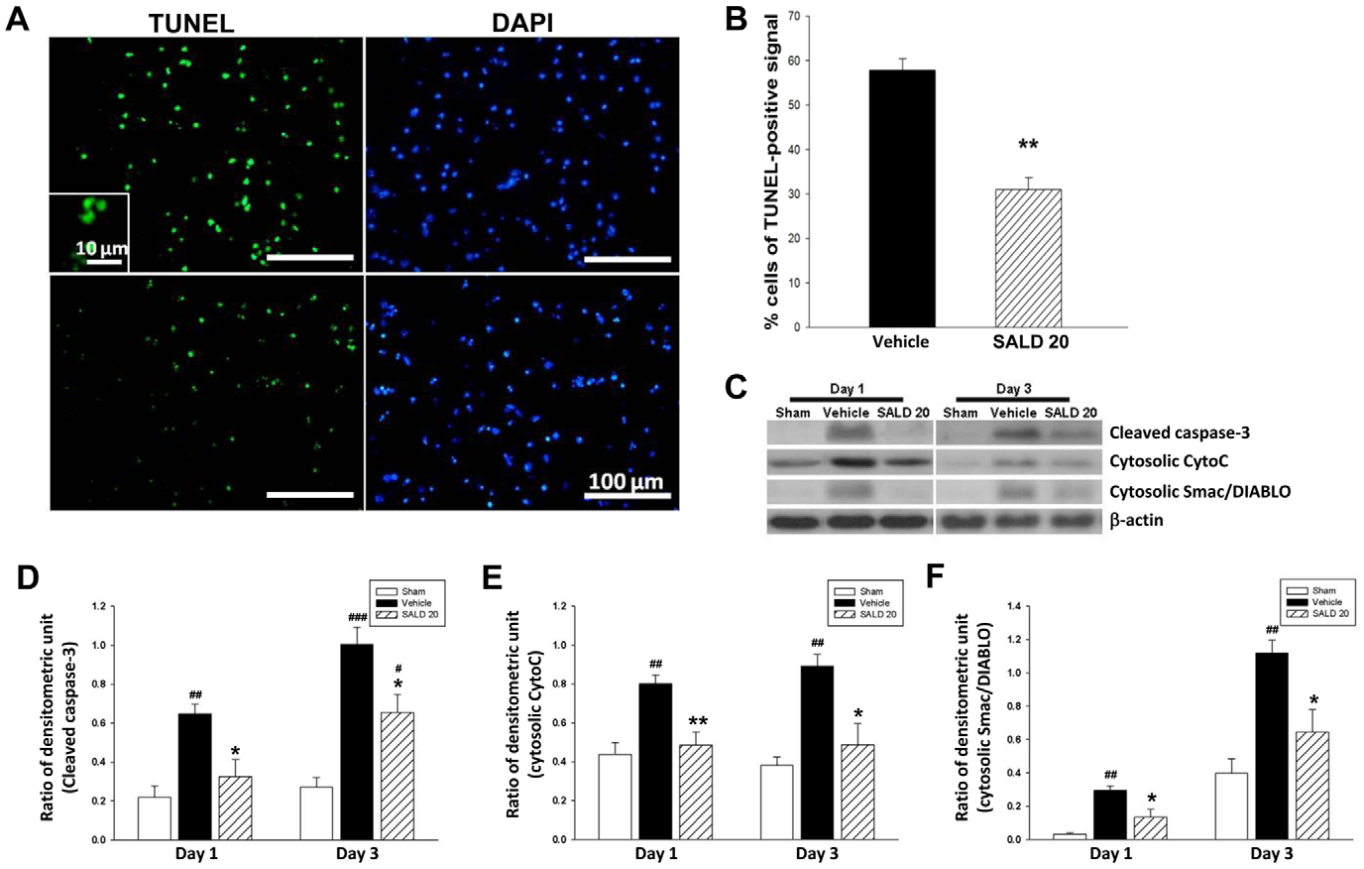
Effects of 20 mg·kg^−1^ salidroside on apoptosis in contusion-injured mice. (**A**) Representative terminal deoxynucleotidyl transferase-mediated dUTP-biotin nick end labeling (TUNEL) staining (green)- and DAPI (blue)-stained brain sections of a 20 mg/kg salidroside (SALD 20)-treated mouse and a vehicle-treated mouse at day 1 post-injury. (**B**) Quantification showed that SALD-treated mice had significantly fewer TUNEL-positive cells around the injured cortical areas at day 1 than observed in the vehicle group. The percentage of TUNEL-positive cells is expressed as the number of TUNEL-stained nuclei/the total number of DAPI-stained nuclei. Sections were stained with DAPI (blue) to show all nuclei. The scale bar is 100 µm. Values are presented as means ± SEM; *P<0.01 versus vehicle-treated injured mice (n = 6 mice/group). (**C**) Representative immunoblots of cleaved caspase-3, cytosolic cytochrome C (CytC) and Smac/DIABLO in the ipsilateral hemisphere from sham-injured, vehicle-treated and SALD 20-treated mice at 1 and 3 days post-injury. Bar graph of densitometric analysis of bands showing a significant decrease of (**D**) cleaved caspase-3 (**E**) cytosolic CytC and (**F**) cytosolic Smac/DIABLO protein levels in the ipsilateral hemisphere of SALD-20 treated mice at 1 and 3 days post-injury, compared with vehicle-treated mice. Values are presented as mean ± SEM; **^##^** *P*<0.01, **^###^** *P*<0.001 versus sham controls, and *P<0.05, **P<0.01 versus vehicle-treated injured mice (n = 6 mice/group at each time point, repeated measures two-way ANOVA).

We next assessed whether SALD 20 reduced post-traumatic apoptosis at days 1 and 3 post-injury. We chose these 2 time-points because previous studies have shown that terminal deoxynucleotidyl transferase-mediated dUTP-biotin nick end labeling (TUNEL) reactivity and apoptotic-related signals peak at 1 day after experimental TBI and last for over 3 days [25]–[28]. TUNEL-positive nuclei were observed in the ipsilateral injured cortex and thalamus but were not seen in the contralateral side at day 1 post-injury (Fig. 4A). SALD 20 treated mice had significantly fewer TUNEL-positive cells around the injured cortical areas at day 1 than that observed in the vehicle group (31.0±6.4% versus 57.8±6.4%; *P*<0.01; Fig. 4B). The reduction of apoptosis was associated with a decrease in cleaved caspase-3, a critical effector caspase of the apoptotic process. Cleaved caspase-3 protein level was low in the sham-operated brains, but it was induced significantly by CCI 1 and 3 days post-injury (*P*<0.01 and 0.001, respectively; Fig. 4C and D). Treatment with SALD 20 significantly decreased the level of cleaved caspase-3 by 49.6% at day 1 (*P*<0.05 versus the vehicle group) and 34.9% at day 3 (*P*<0.05 versus the vehicle group).

To determine whether SALD suppressed activation of caspase-3 by interfering with the release of the mitochondrial inner proteins, CytoC and Smac/DIABLO, from mitochondria, we examined the amount of CytoC and Smac/DIABLO in both mitochondrial and cytosolic fractions. In the sham-operated brains, most CytoC and Smac/DIABLO existed in the mitochondria, with little or no CytoC and Smac/DIABLO located in the cytosol (Fig. 4C). Cytosolic CytoC and Smac/DIABLO were significantly increased in the vehicle group compared to the sham group at 1 and 3 days post-injury, suggesting that mitochondrial permeabilization was induced after CCI (all *P*<0.01; Fig. 4 C, E, and F). SALD 20 significantly attenuated both cytosolic CytoC and Smac/DIABLO levels, which were 60.6% (*P*<0.01) and 45.1% (*P*<0.05), respectively, compared to vehicle-treated injured brains at day 1. These differences remained significant at day 3 when they were 54.7% (*P*<0.05) and 58.1% (*P*<0.05), respectively.

### Salidroside Reduced Apoptosis Via Activation of PI3K/Akt Survival Signaling

**Figure 5:**
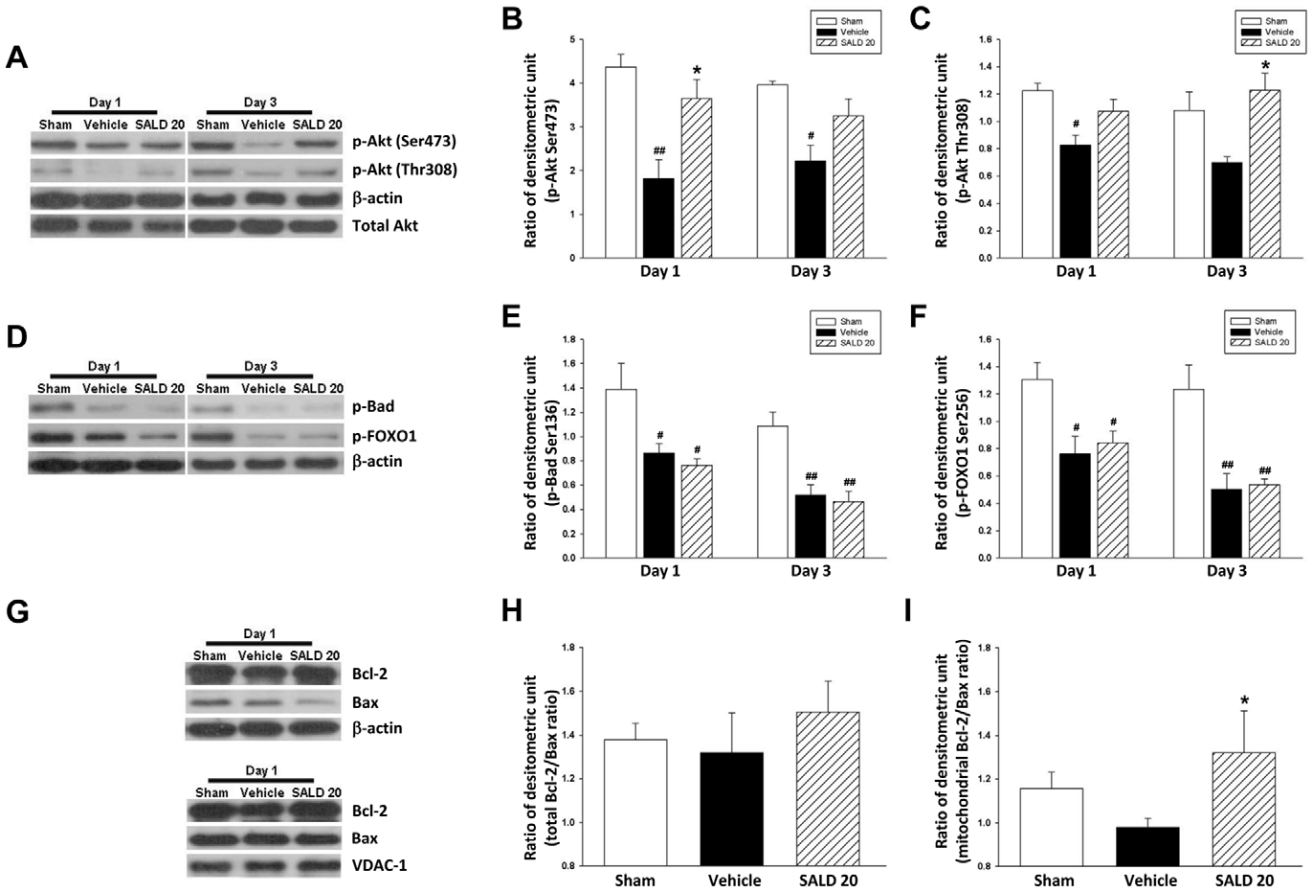
Effects of 20 mg·kg^−1^ salidroside on endogenous survival signals in the cortex of contusion-injured mice. (**A, B, C**) Representative immunoblots and densitometric analysis showed a significant increase in p-Akt Ser473 level in the ipsilateral cortex of 20 mg/kg salidroside (SALD 20)-treated injured mice at day 1 compared with vehicle-treated injured mice. The level of p-Akt Thr308 was increased following SALD 20 treatment at day 3 compared with vehicle-treated injured mice. (**D, E, F**) Representative immunoblots and densitometric analysis showed that there were no differences in the levels of p-Bad and p-FOXO1 in the ipsilateral cortex between the SALD 20 and vehicle groups at day 1 or 3. (**G, H, I**) Representative immunoblots and densitometric analysis showed that the mitochondrial Bcl-2/Bax ratio significantly increased in SALD-20 treated injured mice but no difference was found in the total Bcl-2/Bax ratio. Values are presented as mean ± SEM; **^#^** *P*<0.05, **^##^** *P*<0.01 versus sham controls, and *P<0.05 versus vehicle-treated injured mice (n = 6 mice/group at each time point, repeated measures two-way ANOVA for p-Akt Ser473, p-Akt Thr308, p-Bad, and p-FOXO1 levels; one-way ANOVA for Bcl-2/Bax ratio).

To evaluate whether salidroside-mediated neuroprotection is dependent on antiapoptotic PI3K/Akt signaling, alterations in the protein levels of p-Akt Ser473 and Thr308 were quantified 1 and 3 days after injury. SALD 20 significantly increased p-Akt Ser473 level to 200.7% of the vehicle level at day 1 (*P*<0.05; Fig. 5A and B) and 146.9% of the vehicle level at day 3, even though this difference was not statistically significant (*P*>0.05; Fig. 5A and B). The level of p-Akt Thr308 was also increased to 176.0% of the vehicle level at day 3 after SALD 20 treatment (*P*<0.05; Fig. 5A and C). There was no difference in the levels of p-Bad or p-FOXO1 between the SALD 20 and vehicle groups at either time point (all *P*>0.05; Fig. 5D-F).

Given that the imbalance of proapoptotic Bcl-2 family proteins (e.g., Bax) and antiapoptotic Bcl-2 family proteins (e.g., Bcl-2) can be a primary cause of mitochondrial permeabilization [8], we hypothesized that SALD treatment may reverse the CCI-induced imbalance of Bcl-2 and Bax, both of which are regulated by the PI3K-Akt survival pathway. We analyzed the ratio of Bcl-2 and Bax at both whole cell and mitochondrial levels. At the whole cell level there was no difference in the Bcl-2/Bax ratio between vehicle and sham-operated groups (Fig. 5G and H). Although SALD 20 increased the Bcl-2/Bax ratio slightly, it did not reach statistical significance. The ratio of mitochondrial Bcl-2/Bax decreased slightly by 18.2% following CCI (*P*>0.05; vehicle versus sham groups); however, SALD 20 significantly raised the ratio to 150.6% (*P*<0.05) of the level found in vehicle injured brains (Fig. 5G and I).

**Figure 6:**
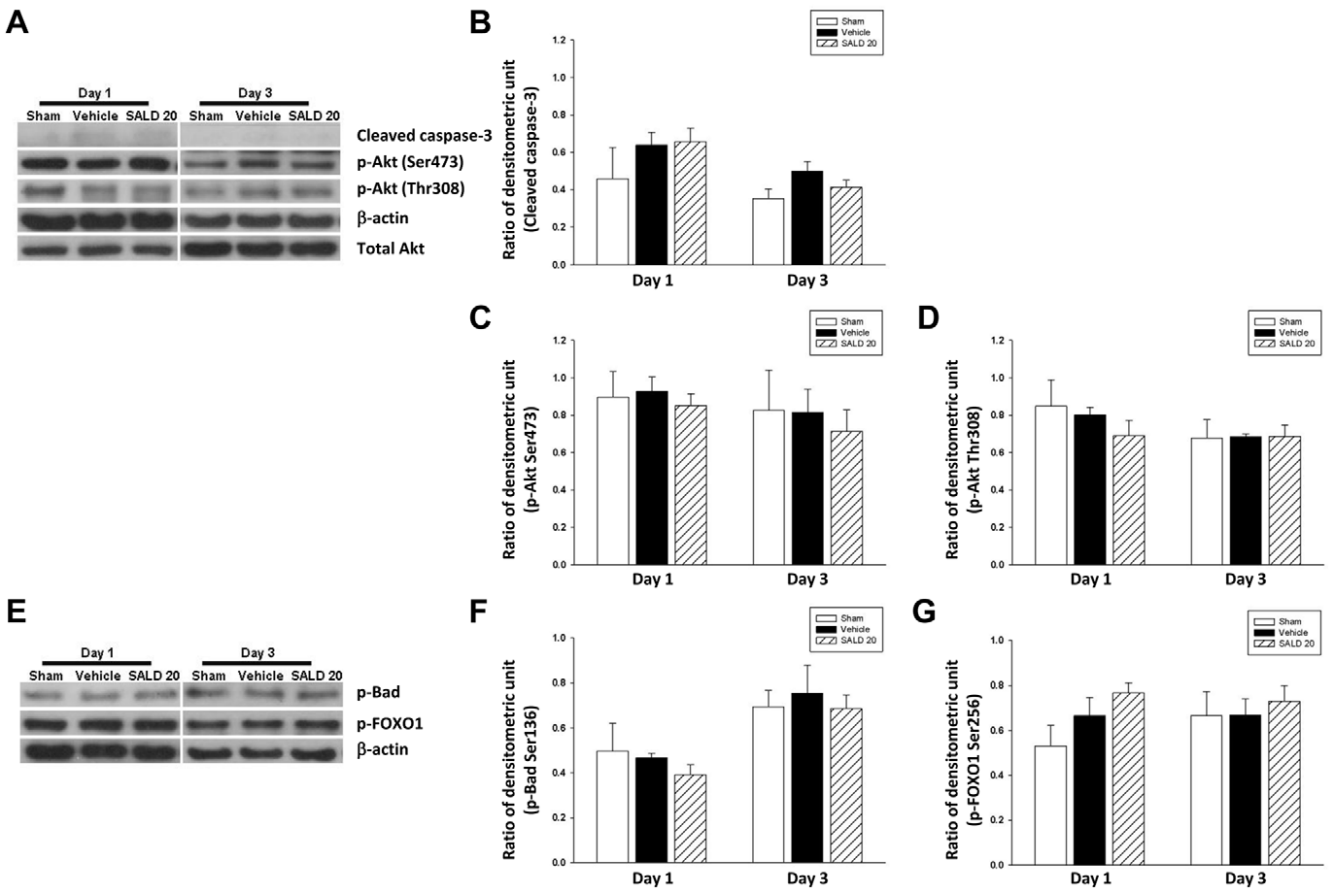
Effects of 20 mg/kg salidroside on apoptotic and survival signals in the hippocampus of injured mice. Representative immunoblots and densitometric analysis showed that (A, B, C, D) levels of cleaved caspase 3, p-Akt Ser473, p-Akt Thr308, (E, F, G) p-Bad and p-FOXO1 in ipsilateral hippocampal samples from injured animals at post-injury day 1 or 3 were not significantly different from those in sham-injured animals (n = 6 mice/group at each time point, repeated measures two-way ANOVA).

We further analyzed the levels of hippocampal apoptotic proteins and survival signals. The levels of cleaved caspase 3, p-Akt Ser473, p-Akt Thr308, p-Bad, and p-FOXO1 in ipsilateral hippocampal samples from injured animals at post-injury days 1 and 3 were not significantly different from those in sham-injured animals (all *P*>0.05; Fig. 6A-G).

### LY294002 Abolished Salidroside-induced Akt Phosphorylation

**Figure 7:**
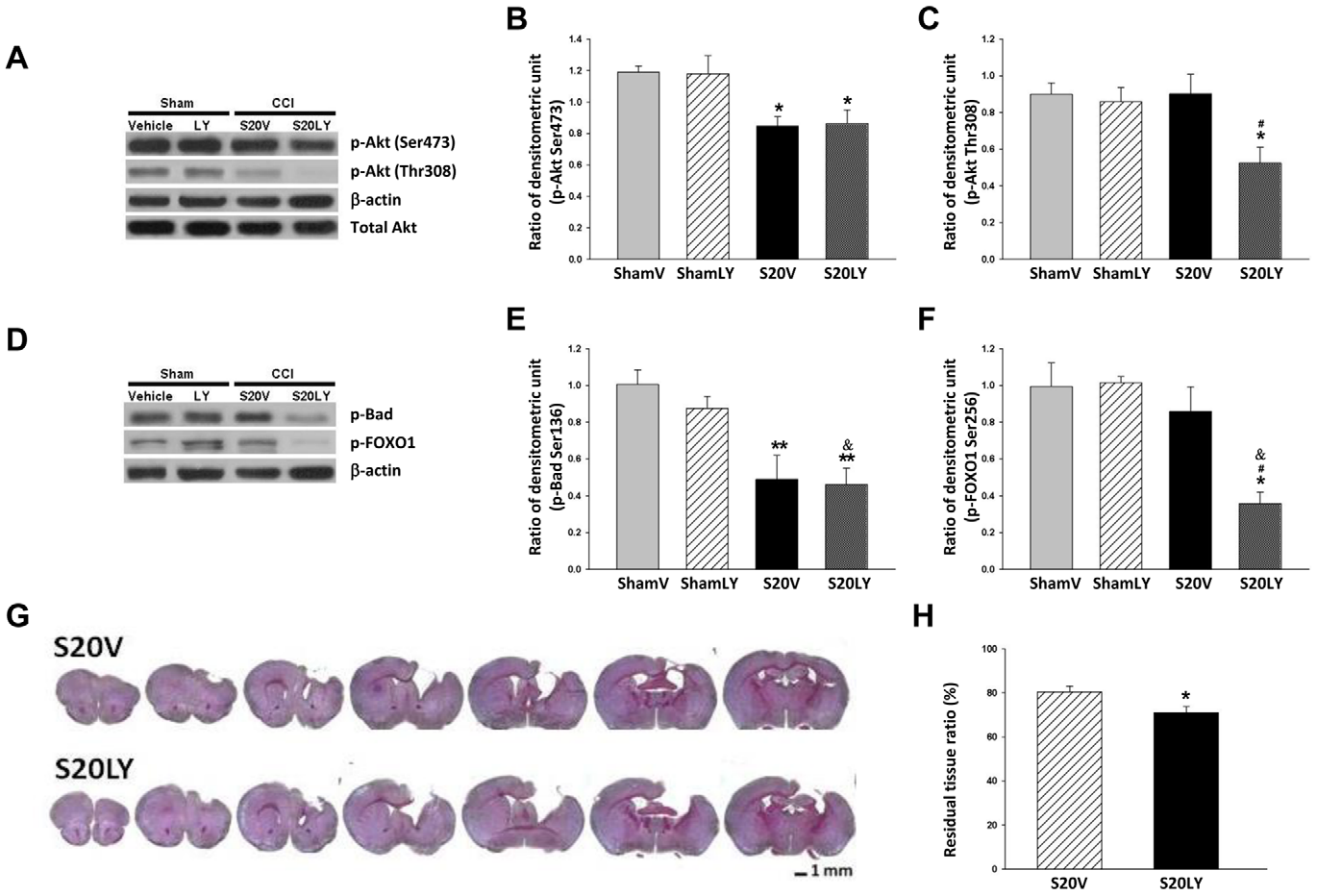
The effects of inhibition of the PI3K/Akt signaling pathway. (**A, B, C, D, E, F**) Representative immunoblots and densitometric analysis in the cortex from sham-injured mice or mice subject to cortical impact injury, infused intracerebroventricular (icv) with saline (vehicle) or LY294002. Mice received either icv pretreatment of LY294002 followed by 20 mg/kg salidroside (SALD 20) after injury (S20Y group) or icv pretreatment of saline followed by SALD 20 after CCI (S20V group). LY294002 did not affect Akt, Bad, or FOXO1 phosphorylation in sham-operated mice, but it significantly abolished SALD 20-induced preservation of p-Akt Thr308 and p-FOXO1 levels in the ipsilateral hemisphere at 1 day post-injury. The levels of p-Akt Ser473 and p-Bad were similar between the S20V and S20LY groups. Values are presented as mean ± SEM; *P<0.05, **P<0.01 versus sham + vehicle (ShamV), and ^&^ *P*<0.05 versus sham + LY294002 (ShamLY), and **^#^** *P*<0.05 versus S20V group (n = 6 mice/group, one-way ANOVA). (**G**) Representative cresyl violet-stained brain sections of S20V and S20LY mice at 28 days post-injury. Scale bar is 1 mm. (**H**) Quantification analysis showed that there was a significant decrease in the residual cerebral tissue ratio in the S20LY group compared with the S20S group. Values are presented as mean ± SEM; *P<0.05 versus S20V group (n = 6 mice/group, Student’s *t*-test).

In order to further confirm the dependence of salidroside-induced neuroprotection on activation of the PI3K/Akt survival pathway, a specific PI3K inhibitor LY294002 was administered to SALD 20-treated CCI mice. LY294002 did not affect Akt, Bad, or FOXO1 phosphorylation in sham-operated mice, but it significantly abolished SALD 20-induced preservation of p-Akt Thr308 (58.1% of the S20V level; *P*<0.05; Fig. 7A and C) and p-FOXO1 levels (41.6% of the S20V level; *P*<0.05; Fig. 7D and F) at day 1 post-injury. Otherwise, the levels of p-Akt Ser473 and p-Bad were similar between the S20V and S20LY groups (both *P*>0.05; Fig. 7A, B, D, and E).

### LY294002 Blocked Salidroside-induced Preservation of Brain Tissue

We then assessed the preservation of brain tissue in CCI mice following the administration of LY294002. The histological analysis showed that there was a significant decrease in the residual cerebral tissue ratio (71.0±6.3%) in the S20LY group compared to the S20S group (80.5±5.8%; *P*<0. 05; Fig. 7 G and H) at day 28 post-injury.

### Delayed Administration of SALD 20 Reduced Neuronal Damage

**Figure 8:**
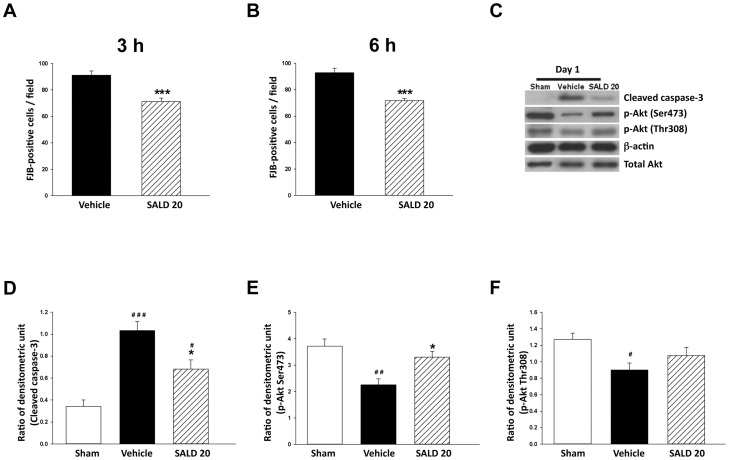
The effect of different lag times of post-treatment with 20mg/kg salidroside. (**A, B**) Mice treated with 20 mg/kg salidroside (SALD 20) had significantly fewer degenerating neurons than vehicle-treated mice in the cortical contusion margin at 1 day when administered at both 3 and 6 h post-injury. The total number of Fluoro-Jade B (FJB)-positive cells is expressed as the mean number per field of view (0.8 mm^2^). (**C, D, E, F**) Representative immunoblots and densitometric analysis showed that SALD 20 treatment significantly decreased cleaved caspase-3 level and increased p-Akt Ser473 level but no significant difference was found in p-Akt Thr 308 level at day 1 when administered at 3 h post-injury. Values are presented as mean ± SEM; **^#^** *P*<0.05, **^##^** *P*<0.01, **^###^** *P*<0.001 versus sham controls, and *P<0.05, ***P<0.001 versus vehicle-treated injured mice (n = 6 mice/group, Student’s *t*-test for FJB staining; one-way ANOVA for immunoblots).

We further performed a delayed treatment study to examine the therapeutic time window of salidroside. SALD 20 treatment significantly reduced the number of FJB-positive neurons around the injured cortical area compared to the vehicle group at day 1 post-injury when administered 3 h (71.2±2.7 versus 91.2±3.3 cells/field; *P*<0.001; Fig. 8A) and 6 h (71.7±1.6 versus 92.9±3.3 cells/field; *P*<0.001; Fig. 8B) after injury. Administration of SALD 20 10 min after injury reduced the number of FJB-positive neurons by 37.3% (Fig. 3D). The effect of treatment initiated at 3 or 6 h post-injury was similar; neuronal damage was reduced by 21.9% when administered 3 h after injury (Fig. 8A) and 22.8% when administered at 6 h (Fig. 8B). We then assessed the levels of apoptotic proteins and survival signals when SALD 20 was administered at 3 h post-injury. SALD 20 treatment significantly decreased cleaved caspase-3 level (Figs. 8C & D) and increased p-Akt Ser473 level (Figs. 8C & E) at day 1 when administered at 3 h post-injury. Although SALD 20 increased p-Akt Thr308 level slightly, it did not reach statistical significance (Figs. 8C & F). Administration of SALD 20 10 min after injury reduced the level of cleaved caspase-3 by 49.6% (Fig. 4D), and increased the levels of p-Akt Ser473 and p-Akt Thr308 by 100.7% (Fig. 5B) and 30.3% (Fig. 5C), respectively, at day 1. Cleaved caspase-3 level was reduced by 34.1%, and the levels of p-Akt Ser473 and p-Akt Thr308 were attenuated by 46.2% and 19.5%, respectively, when SALD 20 was administered 3 h after injury.

### In vitro Studies–neuronal Death is Attenuated by Salidroside in a Concentration Dependent Manner

**Figure 9:**
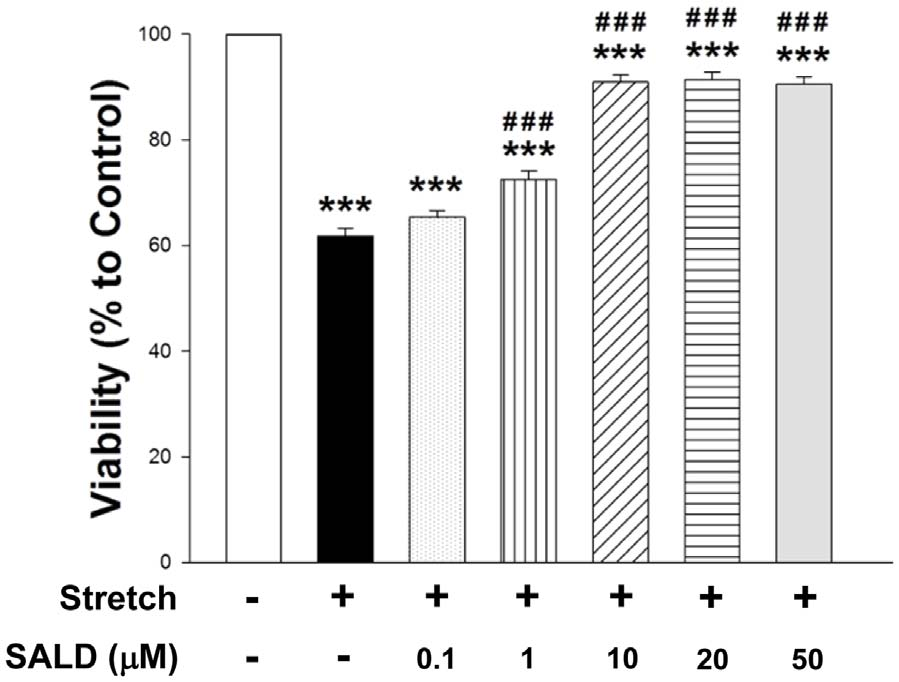
Protective effects of salidroside on stretch-induced cytotoxicity in primary cultured neurons. Treatment with salidroside at different concentrations (0.1, 1, 10, 20, and 50 µM) ameliorated stretch-induced cell viability loss in a dose-dependent manner. Values are presented as mean ± SEM of 3 independent experiments (n = 3); ***P<0.001 versus control, **^###^** *P*<0.001 versus stretch injury alone. All data were representative of 4 independent experiments.

To further extend these *in vivo* findings, a standard model of *in vitro* neuronal stretch injury was used. The 3-[4,5-dimethyl-2-thiazolyl]-2,5-diphenyl-2-tetrazolium bromide (MTT) assay showed that stretch injury significantly induced cell viability loss in cortical neurons (61.8±1.5% of cell viability relative to control; *P*<0.001; Fig. 9). Treatment with SALD at different concentrations (0.1, 1, 10, 20, and 50 µM) ameliorated stretch-induced cell viability loss, restoring the cell survival to 65.3±1.3% (*P*>0.05), 72.5±1.6% (*P*<0. 001), 90.9±1.4% (*P*<0.001), 91.4±1.4% (*P*<0.001), and 90.5±1.4% (*P*<0.001) (relative to vehicle control), respectively, indicating dose-dependent protective effects.

## Discussion

This study showed for the first time that post-injury salidroside treatment significantly attenuated brain tissue damage and long-term behavioral deficits following TBI in mice, and decreased neuronal vulnerability to stretch-induced injury *in vitro*. Salidroside treatment also suppressed apoptosis and preserved mitochondrial integrity. These beneficial effects were associated with increased Akt phosphorylation and mitochondrial Bcl-2/Bax ratio. Co-administration of a specific PI3K inhibitor reversed the reduction of brain tissue loss by salidroside treatment, indicating that its therapeutic action is at least partly mediated by the PI3K/Akt signaling pathway.

We found that single dose salidroside treatment attenuated CCI-induced functional deficits in mice as early as 1 day post-injury and promoted > < sustained improvement of motor-sensory functions over 4-week observation period. This protective effect was associated with a decrease in neuronal damage and apoptotic cell death at day 1, and a reduction in contusion volume and brain tissue loss at day 28. The long-term protective effects in preserving tissue integrity with salidroside treatment is possibly due to a reduction in the number of degenerated neurons detected during the acute phase. Alternatively, some injured but viable neurons may shrink in response to loss of their terminal projections following early axonal injury, and contribute to cerebral atrophy and neurological dysfunctions at the chronic phase [29]. Indeed, increased spacing among remaining neurons occurred in head injury patients with a more severe outcome [30]. Volumetric magnetic resonance imaging (MRI) studies of chronic TBI provides clinical observation for the decrease in volume of grey matter, which is associated with impaired behavioral outcomes in long-term observation [31]–[33]. We noted that long-term preservation of residual tissue was associated with better performances in behavior tasks following salidroside treatment. Our findings suggest that salidroside effectively ameliorated the pathological events leading to post-traumatic deficits during the first 24 h, which consequently led to a better prolonged recovery of neurological function.

We observed that salidroside attenuated brain water content in CCI mice, providing evidence for the effect of salidroside on brain edema after TBI. Cerebral edema has been reported to be one of the major factors leading to the high mortality and morbidity associated with patients with TBI [34]. Cation accumulation is detected in cells undergoing apoptosis, and is induced by apoptotic signaling [35]. This accumulation drives the influx of water into cells and induces cellular swelling, causing cytotoxic edema [34]. In addition, apoptosis enhances inflammation as immune cells are activated by apoptotic cells to clean them up. The cerebral inflammatory response may further damage the microvascular endothelium and lead to blood-brain barrier (BBB) disruption and vasogenic edema [34]. Hence, the anti-edematous effect of salidroside observed in our study is likely to be related to the inhibition of apoptosis.

We found that p-Akt Ser 473 and Thr 308 were constitutively expressed in sham-injured brains, but were diminished 1 day post-injury as apoptotic signatures arose. Both p-Akt Ser 473 and Thr 308 were predominantly localized in neurons post-injury, but rarely found in astrocytes or microglia. These results suggest that the expression of Akt phosphorylation is related to neuronal cell survival after TBI. Previous studies have demonstrated that p-Akt levels are time- and region-dependent after TBI [36]–[38]. This difference may reflect the degree of neuronal damage. Indeed, p-Akt Ser 473 was decreased in the injured cortex of mice after CCI, whereas it was transiently increased in the peri-lesional cortex adjacent to the impact site [39]. This regional difference in Akt activation was also observed following experimental cerebral ischemia [40]. We showed that salidroside treatment increased cortical p-Akt levels and LY294002, a specific PI3K inhibitor, abolished salidroside-induced protection against tissue loss, indicating that the Akt signaling pathway was necessary for the salidroside-induced neuroprotection in this study. Consistent with our findings, several preclinical studies, using various neuroprotective agents, such as growth factors [37], [41], estrogen [42], and statins [43], have reported Akt activation as a potential therapeutic target following TBI. Although pretreated LY294002 reduced p-Akt Thr 308 levels, the p-Akt Ser 473 levels remained unchanged at 1 day post-injury. A reason for this result could be the short half*-*life of LY294002. Since LY294002 was administered 30 min before CCI, the decreased Akt phosphorylation could have been transient (i.e., several hours after treatment).

An unexpected finding of our study was that levels of the downstream targets of Akt kinase activity, p-Bad and p-FOXO1, remained unchanged after salidroside treatment at 1 and 3 days post-injury. Our results are consistent with previous findings in experimental stroke that showed that phosphorylation levels of factors downstream of Akt, such as Bad [44] and GSK3β [45], do not increase together with p-Akt; rather, both of them decrease when p-Akt Ser 473 increases. One possible explanation for these disparities is that Bad and FOXO1 phosphorylation and inhibition could be induced via other signaling pathways, such as MEK/MAPK [46] and SGK3 [47]. Thus, the levels of p-Bad and p-FOXO1 that we measured might may have resulted from activation of other upstream kinases at time points earlier than day 1.

We showed that administration of salidroside decreased cytosolic levels of mitochondrial inner proteins CytoC and Smac/DIABLO and reduced cleavage of caspase-3. This result indicates that salidroside reduced apoptosis at least partially via the mitochondria-dependent endogenous pathway. Mitochondrial integrity is highly controlled, primarily through interactions between pro- and anti-apoptotic members of the Bcl-2 protein family, such as Bcl-2 and Bax [7]. Mitochondrial permeabilization needs Bax activation and its translocation from cytosol onto the mitochondrial surface [7] Our data showed that salidroside treatment attenuated the mitochondrial Bcl-2/Bax ratio, but the ratio remained unchanged in whole cytosol lysate. These findings confirm previous *in vitro* findings that salidroside modulates the balance of apoptosis-regulating proteins and preserves mitochondrial integrity and further demonstrate that this beneficial effect is functional in mouse TBI. However, the beneficial effects of salidroside may be in part attributed to the prevention of free radical and oxidant formation, since salidroside exerts potent antioxidant effects *in vitro*. Further investigations are needed to clarify the anti-oxidative mechanism underlying the salidroside-mediated neuroprotection in TBI.

We observed that a single post-injury injection of 20 mg/kg salidroside protected mice against TBI but did not cause kidney or liver damage. Our present results are consistent with previous reports on animal models of focal cerebral ischemia [21] and Alzheimer’s disease [48]. Nevertheless, previous studies applied prophylactic treatment and only tested the efficacy of salidroside by morphological changes or at a fairly early time point (1 day) after treatment. Our results showing that salidroside administered at 6 h post-injury also exerts a neuroprotective effect is particularly important because it may mean that salidroside could be used clinically for treating TBI. The pharmacokinetic profile of salidroside has not been well established. The mean elimination half-life (t_1/2_) of salidroside following intravenous or oral administration was around 0.5 h and 1.1 h, respectively, in rats [49]. However, the pharmacokinetics in brain tissue or after intraperitoneal administration has not yet been reported. We found that both SALD 20 and SALD 50 were neuroprotective but the SALD50 was less effective than the SALD 20 group. Our results were consistent with those of previous findings showing reduced effectiveness of a higher dose of *Rhodiola. rosea* extract on cognition function of rats [50], [51]. Since salidroside has been demonstrated to enhance immune responses and stimulate cytokine productions both in human peripheral blood lymphocytes [52] and in mice [53], one possibility is that the enhancement of inflammation by salidroside overcomes its anti-apoptotic effect when a higher dose is administered. The reduced effectiveness of a higher dose of salidroside may also be attributed to its sedative effects on the central nervous system [54].

In conclusion, the present study showed that post-injury salidroside administration improved long-term functional and histological outcomes following TBI. This improvement was associated with preservation of mitochondrial integrity and increased Akt activation, and pharmacological PI3K/Akt inhibition reversed the protective effect. Our results suggest that the neuroprotective effects of SALD following TBI are mediated, at least in part, through activation of the PI3K/Akt signaling pathway.

## Materials and Methods

### Animals and Controlled Cortical Impact Injury

All animal procedures were approved by the Animal Research Committee at Cheng Hsin General Hospital (Animal permit number CHIACUC 101–08), and all procedures complied with the Guide for the Care and Use of Laboratory Animals published by the US National Institutes of Health (NIH Publication No. 85–23, revised 1996). Animals were housed under a 12-h light/dark cycle and at constant temperature (21–25°C) and humidity (45–50%). They were allowed free access to pellet chow and water. TBI in mice was induced by a CCI injury model as previously described [55]. Briefly, 8-week-old male C57BL/6 mice (weighing 22–25 g) were anesthetized through the use of intraperitoneal injection of sodium pentobarbital (65 mg/kg; Rhone Merieux, Harlow, UK) and placed in a stereotaxic frame. A 5 mm craniotomy was performed over the right parietal cortex, centered on the coronal suture and 0.1 mm lateral to the sagittal suture, and injury to dura was avoided. Injury was induced using a pneumatic piston with a rounded metal tip (2.5 mm diameter) that was angled 22.5° to the vertical so that the tip was perpendicular with the brain surface at the center of the craniotomy. The impact, with a velocity of 4 ms and a deformation depth of 2 mm below the dura, was applied. The bone flap was immediately replaced and sealed, and the scalp was sutured. Body temperature was monitored throughout the surgery by a rectal probe; temperature was maintained at 37.0±0.5°C using a heated pad. Mice were placed in a heated cage to maintain body temperature, while recovering from anesthesia. Sham-operated mice underwent the same procedure as injured mice, but without CCI.

### Intracerebroventricular Injection

Mice were anesthetized and positioned in a stereotaxic frame. LY294002 (200 nM in 0.5 µL of saline) or equal amount of physiological saline was intracerebroventricularly (icv) injected at 30 min before CCI as previously described [56]. A 1 mm cranial burr hole was drilled into the skull. A 30-gauge needle on a Hamilton syringe was implanted into the lateral ventricle using stereotactic coordinates: 1.5 mm posterior to bregma and 1.0 mm right lateral to the midline, 2 mm in depth. LY294002 or saline was infused into the brain at a rate of 0.05 µL/min over 10 min with an infusion pump (KDS 310, KD Scientific Inc., Holliston, MA, USA), and the needle was left in place for an additional 20 min to prevent reflux. After the needle was removed, CCI procedure was conducted immediately. Sham-operated mice were infused icv with saline, but were not subjected to CCI.

### Experimental Groups and Pharmacological Interventions

Three sets of experiments were performed. The first set was to investigate the protective effects of salidroside. All animals were randomized into 1 of 3 groups (sham injury, CCI + vehicle, CCI + SALD 20). Surgery/injury, behavioral testing, and tissue analyses were performed by different individuals. Behavioral testing and tissue analyses were performed by individuals blinded to the treatment group. Salidroside (Cayman Chemical, Ann Arbor, MI, USA) dissolved in normal saline (0.25 mL) or a corresponding volume of vehicle (saline) was administered intraperitoneally 10 min following injury. Testing after injury was completed as follows: 1) behavioral testing at days 1, 4, 7, 14, 21, and 28 (n = 8–10 mice/group); 2) cresyl violet staining at day 28 (n = 9 mice/group); 3) histology, determination of brain water content, and western blot analysis at day 1 or 3 (n = 5–6 mice/group). To determine the optimal dose of salidroside, a pilot study was performed using 2 different doses (20 and 50 mg/kg) administered 10 min after injury, and neurological deficits and tissue damage were evaluated as the main outcomes. The results showed a significant effect of salidroside at 20 mg/kg, with no further increase in efficacy observed at 50 mg/kg. Therefore, on the basis of these data, a dose of 20 mg/kg was chosen for all subsequent experiments. The second set of experiment was to assess the effect of the inhibition of PI3K/Akt with the specific PI3K inhibitor, LY294002 (Cell Signaling Technology, Danvers, MA, USA), on salidroside effects. Animals were randomized into 4 groups. Mice received either: 1) icv pretreatment of LY294002 followed by SALD 20 after CCI (S20Y group); or 2) icv pretreatment of saline (vehicle control) followed by SALD 20 after CCI (S20V group). 3) Mice were infused icv with saline and were subjected to sham injury. 4) Mice were infused icv with LY294002 and were subjected to sham injury. Testing after injury was completed as follows: 1) cresyl violet staining at day 28 (n = 6 mice/group); 2) western blot analysis at day 1 (n = 6 mice/group). The third set of experiment was performed to examine the neuroprotective time window afforded by salidroside. Mice received intraperitoneal SALD 20 or vehicle (saline) at 3 or 6 h following CCI and were sacrificed 1 day later for FJB staining and western blot analysis (n = 6 mice/group).

### Evaluation of Metabolic Characteristics

Following terminal anesthesia, venous blood was collected via direct right atrial puncture. The obtained blood was centrifuged (3500 rpm for 5 min), and the serum was stored at −20°C until analysis. A chemistry analyzer (Synchron Clinical System LX20; Beckman Coulter, Fullerton, CA, USA*)* was used to measure serum BUN and CRE to assess renal function, and ALT to assess liver function.

### Neurological Function Evaluation

Behavioral testing was performed before CCI and at 1, 4, 7, 14, 21, and 14 days after CCI. The battery of tests consisted of the mNSS, rotarod motor test, and beam walk test. Animals were pretrained for 3 days for the rotarod, and beam walk tests.

### Modified Neurological Severity Score

The mNSS is a composite test of motor, sensory, balance and reflex [57]. One point was recorded for the inability to perform the test or for the lack of a subjected reflex; thus, the higher the score, the more severe the injury. Neurological function was graded on a scale of 0–18 (normal score, 0; maximal deficit score, 18).

### Rotarod

An accelerating rotarod was used to measure motor function and balance [58]. Animals were placed on a 3 cm rotarod cylinder, and the time for which the animals remained on the rotarod was measured. Speed was increased from 6 to 42 rpm every min for a duration of 7 min. A trial ended if the animal fell off the rungs or gripped the cylinder and spun around for 2 consecutive revolutions without attempting to walk on the rungs. One hour before CCI, the mean duration of each animal on the device was recorded with 3 rotarod measurements for the preinjury baseline values.

### Beam Walking

The beam walk test was utilized to evaluate fine motor coordination and function [59]. Mice tended to escape a bright open area by walking along a narrowed wooden beam (0.8×100.0 cm) to enter a darkened goal box at the opposite end of the beam. The latency of the animal to reach the goal box (not to exceed 60 s) was recorded. Three trials were recorded 1 h before CCI as baseline values.

### Brain Water Content

Brain edema was examined by measuring brain water content using the wet−dry/wet brain weight method. Mice were re-anesthetized and decapitated at day 1, a time point associated with maximal edema formation following experimental TBI [22]. Brain water content was measured in a 4mm coronal tissue section of the ipsilateral hemisphere, 2 mm from the frontal pole. Brain samples were immediately weighed on an electric analytical balance to obtain the wet weight and then dried at 100°C for 24 h to obtain the dry weight. The following formula was used for calculation of edema: (wet weight - dry weight)/wet weight ×100%.

### Tissue Processing and Histology

After anesthesia, mice were sacrificed by transcardial perfusion with phosphate-buffered saline (PBS) and then their tissues were fixed with 4% paraformaldehyde at day 1 (for FJB staining, TUNEL, immunofluorescent or cresyl violet histology) and day 28 (for cresyl violet histology) post-injury. Brains were collected and post-fixed in 4% paraformaldehyde overnight and transferred to PBS containing 30% sucrose for cryoprotection. Sections were sliced in a cryostat in 10 µm sections from the level of the olfactory bulbs to the visual cortex.

### Contusion Volume and Residual Cerebral Tissue Analysis

Contusion volumes and residual cerebral tissue ratios were quantified using coronal sections stained with cresyl violet at 20 rostral-caudal levels that were spaced 200 µm apart. Sections were digitized and analyzed using a 1.5× objective and Image J software (Image J, National Institutes of Health, Bethesda, MD, USA). The contusion area was calculated using all cresyl violet-stained sections containing contused brain as previously described [60], and the volume measurement was computed by summation of the areas multiplied by the interslice distance (200 µm). The preservation of cerebral tissue was further evaluated by the ratio of the ipsilateral remaining cerebral hemisphere volume to the volume of corresponding contralateral cerebral hemisphere.

### Fluoro-Jade B Staining

FJB is a polyanionic fluorescein derivative that sensitively and specifically binds to degenerating neurons. Staining was carried out as previously described [24]. Briefly, sections were initially incubated in a solution of 1% NaOH in 80% ethanol for 5 min and then hydrated in graded ethanol (50%, 75%, and 100%; 5 min respectively) and distilled water. Sections were incubated in a solution of 0.06% potassium permanganate for 15 min, rinsed in distilled water for 2 min, and incubated in a 0.001% solution of FJB (Chemicon, Temecula, CA, USA) for 30 min. Sections were observed and photographed under a fluorescence microscope (Olympus BX-51; Olympus, Tokyo, Japan) with blue (450–490 nm) excitation light.

### TUNEL Staining

TUNEL assay was operated using a commercial kit that labels DNA fragmentation with fluorescein isothiocyanate (In situ Cell Death Detection Kit; Roche Molecular Biochemicals, Mannheim, Germany). Sections were first incubated in 25 µg/mL proteinase-K in 10 mmol/L Tris-HCl at 37°C for 15 min. After being washed in distilled water and PBS, sections were incubated in 0.3% hydrogen peroxide solution. Each section was incubated with 70 µL of TUNEL reaction mixture containing terminal deoxynucleotidyl transferase for 60 min at 37°C under humidified conditions. Sections were observed and photographed under a fluorescence microscope (Olympus BX-51) with blue (450–490 nm) excitation light. Negative controls were obtained by omission of terminal deoxynucleotidyl transferase.

### Immunofluorescent Staining

Immunofluorescent staining was carried out to assess the cellular localization of p-Akt (Ser473 and Thr308). Double immunofluorescence labeling was performed by simultaneous incubation of anti–p-Akt Ser473 or Thr308 (Table 2) with anti-neuronal nuclei antigen (neuron marker), anti-F4/80 (microglia marker), and anti-glial fibrillary acidic protein (astrocyte marker). Sections were incubated overnight at 4°C with anti–p-Akt (Ser473 or Thr308) antibody plus one of the antibodies to a specific cellular marker. Sections were then washed and incubated with Alexa Fluor 488 or Alexa Fluor 594 (1∶400; Molecular Probes, Eugene, OR, USA) for 2 h. All sections were observed and photographed under a fluorescence microscope (Olympus BX-51).

**Table 2:** Antibodies used in immunofluorescence and western blot.

Primary antibody	Commercial source	Catalog number	Species	Antibody type	Concentration
p-AKT (Ser472)	Cell signaling	9271	Rabbit	Polyclonal	WB 1∶2000
					IF 1∶400
p-AKT (Thr308)	Cell signaling	4056	Rabbit	Monoclonal	WB 1∶1000
AKT	Cell signaling	9272	Rabbit	Polyclonal	WB 1∶2000
Active caspase 3	Cell signaling	9661	Rabbit	Polyclonal	WB 1∶1000
Bcl-2	Santa Cruz	Sc-492	Rabbit	Polyclonal	WB 1∶5000
Bax	Santa Cruz	Sc-493	Rabbit	Polyclonal	WB 1∶1000
p-Bad (Ser136)	Cell signaling	4366	Rabbit	Monoclonal	WB 1∶1000
p-FOXO1 (Ser256)	Cell signaling	9461	Rabbit	Polyclonal	WB 1∶1000
Cytochrome C	Upstate	556433	Mouse	Monoclonal	WB 1∶1000
Smac/DIABLO	Abcam	ab9709	Rabbit	Monoclonal	WB 1∶500
GFAP	Invitrogen	13-0300	Rat	Monoclonal	IF 1∶200
NeuN	Millipore	MAB377	Mouse	Monoclonal	IF 1∶500
F4/80	Serotec	MCA497GA	Rat	Monoclonal	IF 1∶100

### Quantification of Fluoro-Jade B and TUNEL Staining

FJB and TUNEL staining was quantified on stained sections from the injury core at the level 0.74 mm from the bregma. Three sections per animal were photographed by a microscope. FJB- and TUNEL-positive cells were counted by sampling an area of 920×860 µm^2^ in 3 randomly selected, non-overlapping fields with the magnification of 200. The total number of FJB-positive cells was expressed as the mean number per field of view. Quantification of TUNEL staining was expressed as the percentage of nuclei that were stained by the TUNEL assay divided by the total number of DAPI–stained nuclei.

### Protein Extraction and Western Blot

Brains of injured or sham animals were collected 1 day and 3 days post CCI or sham surgery, and a 4 mm coronal section from the injured area over the parietal cortex and ipsilateral hippocampus were collected. The protein samples were obtained from tissue homogenates in 300 µL of ice-cold reagent (T-PER reagent; Pierce Biotechnology, Rockford, IL, USA) with a complete mini protease inhibitor cocktail (Roche Molecular Biochemicals) centrifuged at 14000*g* for 30 min. Protein concentration was determined by Bradford reagent spectrophotometrically at A595. Equal amounts of protein samples were denatured in gel-loading buffer at 100°C for 5 min, and then loaded onto sodium dodecyl sulfate-polyacrylamide gels to be separated. Separated proteins were transferred to Immobilon-P membranes (Millipore, Billerica, MA, USA), blocked with the use of 5% milk in PBST and probed with primary antibodies. Afterward, the membranes were washed and incubated with anti-rabbit or anti-mouse horseradish peroxidase–linked secondary antibodies (Santa Cruz Biotechnology, Santa Cruz, CA, USA; 1∶1000) at 4°C for 1 h. The relative intensity of protein signals was normalized to the corresponding β-actin intensity and was quantified by densitometric analysis with the use of Image J software.

### Isolation of Mitochondria

Dissected cortices (prepared as in western blot analysis) were immediately homogenized in 300 µL of ice-cold cytosol extraction buffer (Cytosol/Mitochondria Fractionation kit; Merck, Rockland, Massachusetts, USA) with a protease inhibitor cocktail and DTT. The homogenates were then centrifuged at 700 *g* for 10 min at 4°C, and the supernatant was further centrifuged at 10000 *g* for 30 min at 4°C. The 10000 g supernatant was collected as the cytosolic fraction, and the pellet contained the mitochondria. The mitochondrial fraction was resuspended in 50 µL mitochondrial extraction buffer mix containing protease inhibitors and DTT for 10 s and saved as mitochondrial fraction, or maintained intact in PBS at –80°C until use.

### Primary Cortical Neuron Culture, Stretching of Neurons and Assessment of Cell Viability

All culture medium supplies were from Invitrogen (Invitrogen, Carlsbad, CA, USA). Cortical neuronal cells were prepared from embryonic day 15.5 C57BL/6 mice and cultured in serum-free conditions as previously described [61]. Briefly, the cortices were isolated and digested in 0.5 mg/mL papain at 37°C for 15 min, followed by dissociation in Hibernate-A medium (containing B27 supplement) by aspirating trituration. The cells were then plated onto poly-L-lysine-coated 6-well silastic culture plates (Flexcell International Corporation, Hillsborough, NC, USA) at a density of 5×10^4^ cells/cm^2^ and maintained in Neurobasal medium supplemented with B27, 10 units/mL penicillin, 10 mg/mL streptomycin, and 0.5 mg/mL glutamine. Four days after plating, 50% of the medium was removed and replaced with fresh medium. The cells were cultured at 37°C in a humidified tri-gas incubator (5%O_2_, 90% N_2_, and 5% CO_2_) and were used at day 10 *in vitro*. The cortical cultures contain mostly neurons and there were <2% of glia cells, as determined by immunohistochemical staining.

Cultures were stretched by rapid deformation of the silastic culture plates (Flexplates), using the Cell Injury Controller II (Custom Design and Fabrication; Virginia Commonwealth University, Richmond, VA, USA) as previously described [62]. This device uses a regulated flow of compressed gas to rapidly pressurize cells cultured on flexible substrates, causing a radial stretch injury. This combination of pressure and biaxial stretch has been demonstrated to correlate with traumatic injury *in vivo* [62]. Briefly, the injury controller delivered two 50-ms pulses (28 psi) of compressed nitrogen, which resulted in a 10.2 psi peak pressure to the well (membrane deformation 6.5 mm). This stimulus intensity corresponds to the “moderate stretch” [62]. The primary cultured neurons were treated with 0.1, 1, 10, 20, and 50 µM salidroside, respectively, immediately following injury. The cortical neurons cultured in plain medium served as control. Cell viability analysis was performed 24 h after injury.

The cell viability was determined by assessing the degree of MTT reduction. Briefly, cells were incubated at 37°C for 2 h with MTT (0.5 mg/mL final concentration; Sigma-Aldrich; St. Louis, MO, USA). Afterwards, a solution of anhydrous isopropanol, HCl (0.1 N), and 0.1% Triton X-100 was added for the dissolution of formazan crystal. The optical density was determined at 570 nm using a microplate reader. Cell viability was expressed as a percentage of the control culture. The experiments were repeated 4 times with different batches of primary cultures.

### Statistical Analysis

Data are presented as the mean ± standard error of the mean (SEM). For comparisons among multiple groups, one-way or two-way analysis of variance (ANOVA), followed by post-hoc (Bonferroni) test, was used to determine significant differences. Differences between 2 groups were tested using the Student’s *t*-test. Statistical significance was set at *P*<0.05.
